# A simulation-based randomized trial of ABCDE style cognitive aid for emergency medical services CHecklist In Prehospital Settings: the CHIPS-study

**DOI:** 10.1186/s13049-023-01144-3

**Published:** 2023-11-17

**Authors:** Helena Droege, Heiko Trentzsch, Alexandra Zech, Stephan Prückner, Sebastian Imach

**Affiliations:** 1 Department of Trauma and Orthopedic Surgery, Cologne-Merheim Medical Center (CMMC), University Witten/Herdecke, Ostmerheimer Str. 200, 51429 Cologne, Germany; 2 Department of Orthopedics and Traumatology, University Medical Center of the Johannes Gutenberg University, Langenbeckstr. 1, 55131 Mainz, Germany; 3 Institut Für Notfallmedizin Und Medizinmanagement (INM), Klinikum der Universität München, LMU München, Schillerstr. 53, 80336 Munich, Germany

**Keywords:** Checklists, ABCDE scheme, ABCDE mnemonic, Return of spontaneous circulation (ROSC), Out of hospital cardiac arrest (OHCA), Resuscitation, Medical simulation, Patient safety

## Abstract

**Background:**

Checklists are a powerful tool for reduction of mortality and morbidity. Checklists structure complex processes in a reproducible manner, optimize team interaction, and prevent errors related to human factors. Despite wide dissemination of the checklist, effects of checklist use in the prehospital emergency medicine are currently unclear. The aim of the study was to demonstrate that participants achieve higher adherence to guideline-recommended actions, manage the scenario more time-efficient, and thirdly demonstrate better adherence to the ABCDE-compliant workflow in a simulated ROSC situation.

**Methods:**

CHIPS was a prospective randomized case–control study. Professional emergency medical service teams were asked to perform cardiopulmonary resuscitation on an adult high-fidelity patient simulator achieving ROSC. The intervention group used a checklist which transferred the ERC guideline statements of ROSC into the structure of the ‘ABCDE’ mnemonic. Guideline adherence (performance score, PS), utilization of process time (items/minute) and workflow were measured by analyzing continuous A/V recordings of the simulation. Pre- and post-questionnaires addressing demographics and relevance of the checklist were recorded. Effect sizes were determined by calculating Cohen’s *d*. The level of significance was defined at *p* < 0.05.

**Results:**

Twenty scenarios in the intervention group (INT) and twenty-one in the control group (CON) were evaluated. The average time of use of the checklist (CU) in the INT was 6.32 min (2.39–9.18 min; SD = 2.08 min). Mean PS of INT was significantly higher than CON, with a strong effect size (*p* = 0.001, *d* = 0.935). In the INT, significantly more items were completed per minute of scenario duration (INT, 1.48 items/min; CON, 1.15 items/min, difference: 0.33/min (25%), *p* = 0.001), showing a large effect size (*d* = 1.11). The workflow did not significantly differ between the groups (*p* = 0.079), although a medium effect size was shown (*d* = 0.563) with the tendency of the CON group deviating stronger from the ABCDE than the INT.

**Conclusion:**

Checklists can have positive effects on outcome in the prehospital setting by significantly facilitates adherence to guidelines. Checklist use may be time-effective in the prehospital setting. Checklists based on the ‘ABCDE’ mnemonic can be used according to the ‘do verify’ approach. Team Time Outs are recommended to start and finish checklists.

## Background

Human error is a leading cause of death in modern patient care. The estimated number of deaths due to medical errors in registered hospital admissions in the USA is 400,000 deaths/year [1]. In 2000, the report 'To Err Is Human: Building a Safer Health System' estimated that 44,000 people die annually in the USA due to medical errors [2]. Adverse events i.e. iatrogenic injuries occur in 3.7% of all hospitalizations, a relevant portion (27.6%) of adverse events were due to negligence [3]. Potentially, a large proportion of these human errors are preventable. Since many medical procedures rely on perfect memory while humans are prone to short term memory loss [4]. Standardization followed by implementation of consistently identical process flows, is a suitable measure of reducing these errors. Checklists are a powerful tool for standardization of processes.

Individualized and sufficiently implemented cognitive aids like checklists increase performance when solving time-critical and challenging medical emergencies mainly by reducing omissions [5–7]. Checklists allow complex processes to be structured in a reproducible manner, optimize team interaction, and prevent errors related to human factors (e.g., memory lapses due to task overload) [8]. In addition, they help to ensure that the content of processes—the medical therapy- is complete and up-to-date, which can be measured, for instance by compliance with valid guidelines. Simultaneously, they can accelerate processes. They are, therefore, a powerful tool for minimizing human performance limitations.

Checklists have long been successfully used in high reliability organizations (HRO) such as nuclear power production, military operations and aviation [9]. The perform hazardous and complex operations with demand of a very low failure rate [10]. In aviation, they are used routinely, including digital formats, as a control tool for safe flight preparation, execution, and follow-up as part of the prevailing safety culture [11].

In medicine, checklists are used in the operating room. The ‘WHO Surgical Safety Checklist’ uses perioperative timeouts at three defined time points (before induction of anesthesia, directly before the skin incision, and before the patient leaves the operating theatre) to check safety-relevant items for the entire surgical team. In a prospective randomized study, the WHO Surgical Safety Checklist achieved a significant reduction in the overall complication rate at all study sites from 11 to 7% (*p* < 0.001). The rates of postoperative infections and unplanned revision surgeries decreased significantly. The overall hospital death rate was reduced from 1.5 to 0.8% by the WHO Surgical Safety Checklist. Therefore, the checklist is used by approximately 2,500 hospitals worldwide today [12, 13]. In the intensive care setting, a checklist can ensure compliance with preventive measures against catheter-associated infections when central venous catheters are inserted, thereby significantly reducing the incidence of bloodstream infections at 3 months after implementation (2.7 infections/1000 catheter days to 0, *p* = 0.002) [14]. In the prehospital setting of emergency medicine checklists can improve documented adherence to treatment guidelines and can especially amplify the use of relevant pharmacological interventions. While not relevantly restricted in the personal medical practice users are not regularly experiencing an individual benefit of checklist use [15]. Finally checklists have a value during the resuscitation training of health care professionals. They either support by directly conveying medical content or enabling a structured evaluation of the training goals [16].

While not mentioning “checklist” or “cognitive aid” in the adult advanced life support section current European resuscitation guidelines encourage the use of safety checklists to minimise human factors during medical procedures or special circumstances [17, 18].

In principle, the use of checklists in medicine allows that the treatment of the patient does not exclusively depend on human memory.

For the design of a checklist for the pre-hospital setting a suitable structure and proper methods of usage must be identified. The ‘ABCDE’ mnemonic is an logical choice for the structure as it offers a well-defined linear flow, provides some simplification to the process, the provider have a comprehensive amount of experience at the “sharp-end” use and finally its effects on prehospital treatment time and workflow during trauma resuscitation are proven [9, 19].

Two basic concepts are available for the usage of checklists. The ‘challenge & response’ approach, or ‘cookbook’ approach, takes place between two users, whereby one user calls out an item in the exact order of the checklist (challenge) and the second user executes the item directly under the control of the first user (response). This results in a high level of application security at the expense of an inflexible action framework that is prone to failure. A ‘do verify’ approach is a more flexible method in which the team first works through the known contents of the checklist and then checks the completeness of the checklist during a timeout and completes it if necessary (verify) [20, 21].

Prehospital checklists can increase patient safety and outcome by facilitating adherence of prehospital therapy to currently valid therapy guidelines (i.e. by the European Resuscitation Council [ERC]). For our study, the guideline content of the therapy of return of spontaneous circulation (ROSC) after cardiopulmonary resuscitation was transferred into a prehospital checklist in the form of the ‘ABCDE’ mnemonic. The 2010 version of the ERC guidelines was the first to specifically address this issue and to define complex standards of care. Therapeutic hypothermia (nowadays: temperature target management) was included as a concept in the guideline for the first time and could, therefore, be a surrogate for the rapid transfer of knowledge through checklists [22–24].

The aim of the study was to demonstrate that professional emergency medical service (EMS) teams working through a simulated ROSC situation using a checklist based on the ‘ABCDE’ mnemonic firstly achieve higher adherence to guideline-recommended actions, secondly manage the scenario more time-efficient, and thirdly demonstrate better adherence to the ABCDE-compliant workflow than EMS teams working without checklist support.

## Methods

### Study design/population

This was a prospective randomized case–control study (CHIPS, checklist in prehospital settings, German Clinical Trials Register Study ID 00005156). We used the reproducible setting of a full-scale medical simulation. The simulations took place at the Human Simulation Center (HSC) of the University Hospital Munich’s Institute for Emergency Medicine and Medical Management.

The study participants were professional EMS providers who had completed training according to federal regulations and an active assignment with an EMS. The study participants were recruited from all 26 ambulance service areas in the state of Bavaria (Germany). Each study scenario was performed by a team of four participants typical of the German prehospital setting (two paramedics representing ambulances of type C [Mobile Intensive Care Unit, Rettungswagen], one pre-hospital emergency physician and one paramedic together representing a typical German physician staffed emergency unit [NEF]).

**Fig. 1:**
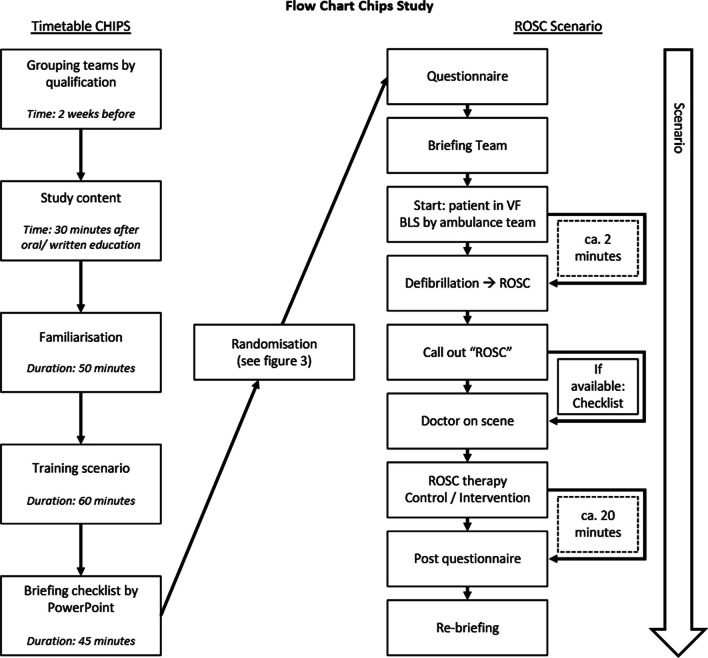
Flow chart of data collection in the CHIPS study. *EMS*: emergency medical services, *HSC*: Human Simulation Center, *CHIPS*: Checklist in prehospital settings, *ROSC*: Return of spontaneous circulation, *BLS*: Basic Life Support, *NEF*: Notarzteinsatzfahrzeug (emergency vehicle)

Figure 1 shows the chronological sequence of data acquisition in the CHIPS study.

The ethics committee of the Faculty of Medicine of the Ludwig-Maximilians-Universität in Munich (ID 475-12) and the University of Witten/Herdecke, Germany approved the study (ID 12/2020). All participants provided written informed consent before participation.

### Simulation scenario

The scenario took place inside a standard ambulance vehicle (Typ C) mock-up at HSC. Participants were asked to perform cardiopulmonary resuscitation on an adult high-fidelity patient simulator (SimMan 3G, Laerdal Medical AS; Stavanger, Norway). It was reported that he suffered from witnessed cardiac arrest with ventricular fibrillation (see scenario script in supplements). At the time of the study, the 2010 ERC guidelines were well established (01/13 to 06/14). ROSC established following the third defibrillation. After ROSC occurred the prehospital emergency physician arrived on scene with a third paramedic.

Before the start of the scenario, an HSC instructor provided a standardized introduction to the patient simulator, simulation environment, and equipment. Teams used an automated external defibrillator in the semi-automatic mode (LIFEPAK 15 defibrillator, Physio-Control; Redmond, WA, USA). The device was adjusted to cprMAX mode, a technology intended to minimize hands-off times while charging. The team indicated that they used this configuration in daily routines.

### Checklist

**Fig. 2:**
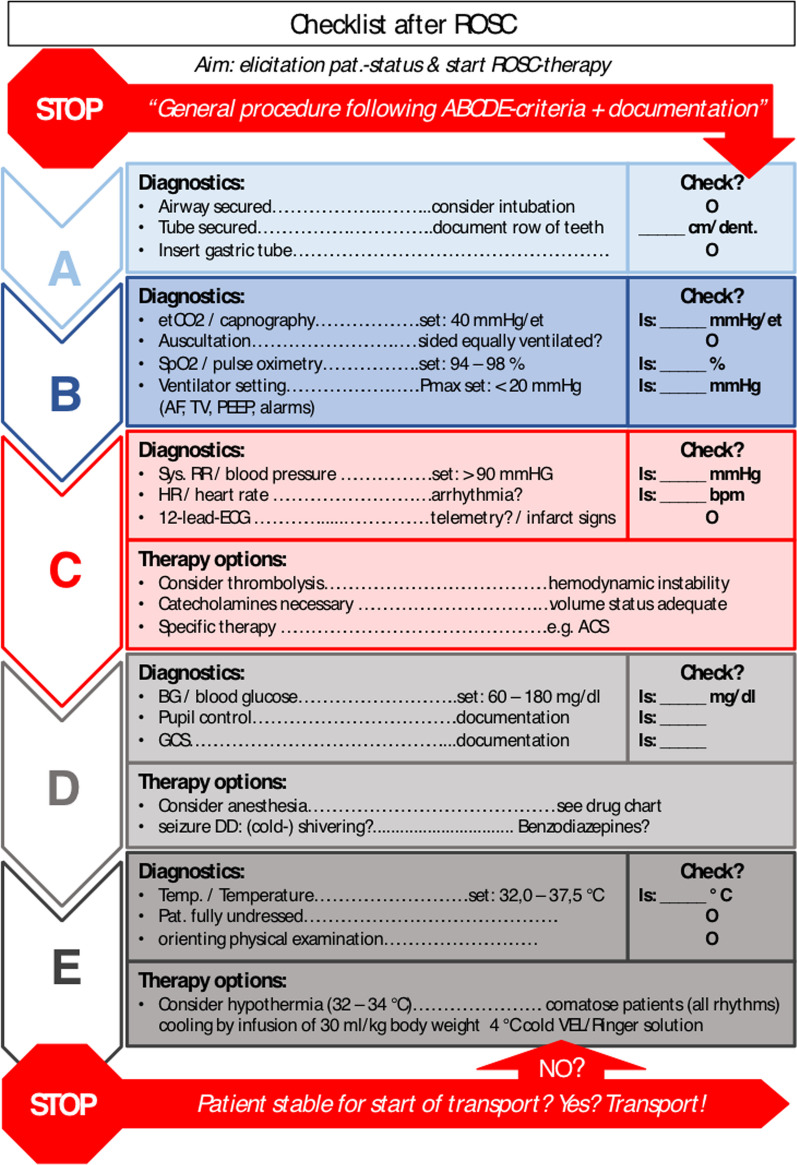
Checklist based on ABCDE mnemonic. **A**: Airway, **B**: Breathing, **C**: Circulation, **D**: Disability

The checklist used in the scenario transferred the guideline statements of the ERC Consensus 2010 on the management of patients with ROSC into the structure of the well-established, prehospital ‘ABCDE’ mnemonic, which should enable priority-oriented care of emergency. The five sections are organized according to the ‘treat first what kills first’ principle. The sections are: ‘Airway’ (A), with four items; ‘Breathing’ (B), with four items; ‘Circulation’ (C), with seven items; ‘Disability’ (D), with six items; and ‘Exposure’ (E), with four items (Fig. 2). The graphical representation of the checklist was prepared following the recommendations for the design of medical checklists formulated by Verdaasdonk et al. [20]. The checklist was recommended to start with a standardized team timeout according to Rall et al. [25] For the teams in the intervention group (INT), the checklist was released by announcement via speaker after the teams verbalized the ROSC situation (Fig. 1 Flow Chart CHIPS-Study). Individual time periods of checklist use in the intervention scenario were measured and summed up (checklist use duration). The teams received no instructions on how to use the checklist. Style of checklist use and other findings concerning the checklist were also recorded as additional findings.

### Performance score (PS)

To allow assessment of guideline adherence, we created the so-called Performance Score (PS) as an evaluation measure. This score evaluates the completeness and prehospital relevance of guideline recommendations for ROSC therapy based on an expert consensus (medical directors of the emergency medical services of the state of Bavaria). Therefore, a numerical value was assigned to each of 25 guideline statement in order to represent the level of obligation defined by the expert consensus. Statements termed with ‘*may*’ got one point, ‘should’ statements correspond to two points and ‘must’ is equal to 3 points. The individual guideline recommendation was multiplied by this numerical value and weighted accordingly.

A maximum PS of 62 points could be achieved, since the experts evaluated 18 guideline statements (GS) with three points, three GS with one point and three GS with zero points. The target temperature management (TTM), which was recommended for the first time in the guideline consensus in 2010, was the only item to receive a value of 5, as it was intended to serve as a surrogate parameter for the speed of knowledge transfer from the guideline to practical clinical application.

### Observation time

To ensure study continuation on time, an indefinite observation period could not be realized for ROSC therapy section. After the call-out of ROSC, each scenario was continued for 10 min according to protocol. During this time, the patient remained unconscious.

To assess time management and effective use of procedure time, the number of processed items per minute of observation was measured (items/min).

### Workflow

To assess the degree of organization of the work process, compliance with the defined sequence of actions specified by the checklist was examined. Each item was assigned a fixed place according to the ‘ABCDE’ mnemonic as it was indicated by the checklist. The score is the sum of the deviations from the fixed order divided by the number of items performed.

### Data collection/analysis

We captured the following data: (a) continuous A/V recordings of the simulation scenario from different viewing angles and (b) real-time vital sign data from the patient’s monitor using the picture-in-picture technology. The participants also completed a standardized questionnaire with demographic data (before scenario) and rated the significance of the simulation scenario for their daily practice (right after scenario). The raw video data of 2 scenarios were damaged and could not be evaluated. The corresponding pre- and post-questionnaires of these scenarios could not be removed due to anonymization.

Statistical analyses were performed using SPSS statistical software (version 27; IBM Inc., Armonk, NY, USA), and Microsoft Excel and Office 365 (Microsoft, Redmond, WA, USA) were used. Data are expressed as absolute and relative values or means ± standard deviations (SD). In case of skew distributed data, the median (interquartile range) was used instead of the mean.

Effect sizes were determined by calculating Cohen’s *d*, with a Cohen’s *d* of 0.2, 0.5, and 0.8 indicating small, medium, and large effects, respectively.

The *t*-test was applied to show the mean differences between the two groups. The correlation determination of the variables was calculated using the determination of Pearson correlation. The level of significance was defined at *p* < 0.05.

## Results

**Fig. 3:**
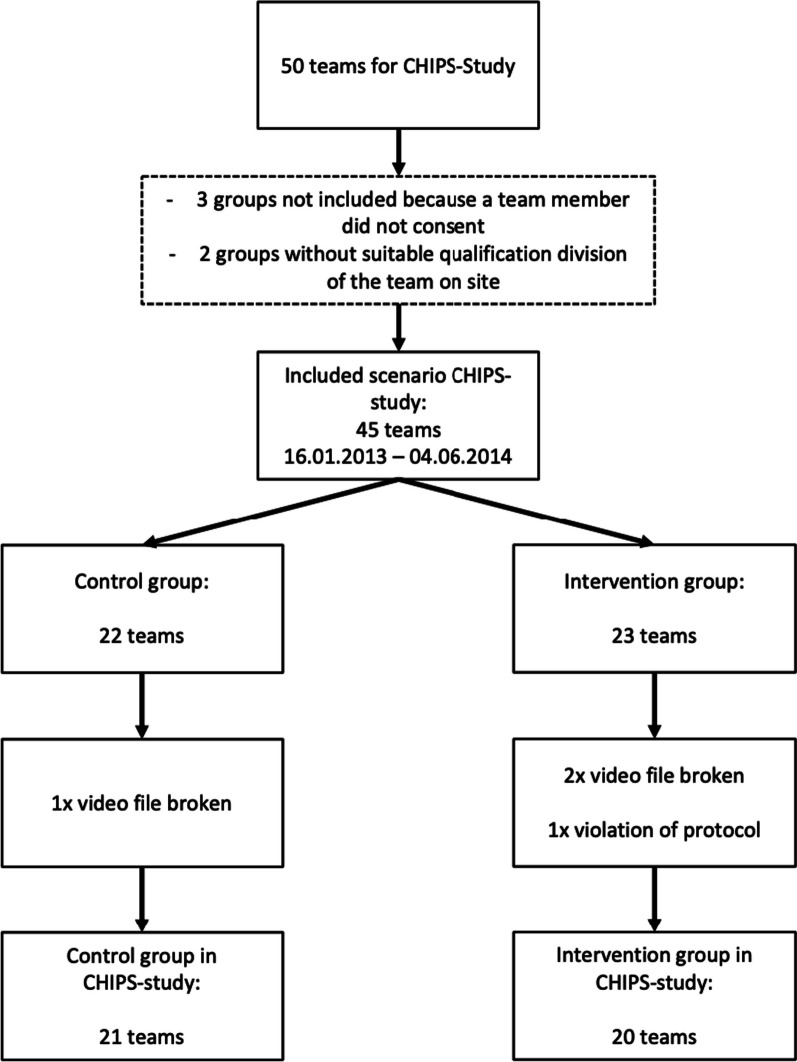
Sample Size CHIPS-Study. CHIPS: Checklists in prehospital settings

Twenty scenarios in the INT and twenty-one in the CON could be evaluated (Fig. 3). The scenario durations showed no significant differences (INT, 10.24 ± 0.52 min; CON, 10.10 ± 0.33 min; *p* = 0.305; 95% confidence interval [CI] − 0.42 to 0.13 min).

Participants (n = 172, 30 women, 122 men, 20 not specified) represented 23 of the 26 emergency service areas at study time. All emergency physicians were board-certified, while 9.8% (12/123) of the paramedics were still in training. About two thirds (61.0%) of the participants had over 10 years of professional experience. In both groups, just under half of the participants had experienced ROSC between one and three times within 1 year before study entry (INT: 43/92, 47.0%; CON 39/80, 49.0%). Approximately one quarter of the participants in both groups had experienced ROSC four to six times (INT: 20/92, 22.0%; CON: 19/80, 24.0%).

In the post-scenario questionnaire both groups equally perceived the scenario as realistic on a 6-point Likert scale (INT: 4.97, CON: 5.06, *p* = 0,582) and were satisfied with the individual performance in the scenario from a medical perspective (INT: 4.43, CON: 4.67, *p* = 0.163).

A timeout at the beginning of the ROSC treatment (study period) was performed by 21 teams in the CON and 18 in the INT. On average, the timeout lasted 0.51 min in the CON and 1.40 min in the INT. The difference was not statistically significant (*p* = 0.146).

### Guideline adherence according to performance score

The results are displayed in Table 1. Mean PS of INT was significantly higher than CON, with a strong effect size (*d* = 0.935). Further analysis of the individual subsections of the checklist showed a significantly higher PS for the INT concerning the subsection ‘Airway’ (A), ‘Breathing’ (B) and ‘Environment’ (E).

**Table 1:** Guideline adherence according to performance score

	Maximum score	Intervention group (INT): mean (standard deviation) min–max	Control group (CON): mean (standard deviation). Min–max	Degrees of freedom (df)	T	p	Effect size Cohen´s d
Performance Score (PS)	62	39.10 (9.04) 18–59	31.33 (7.53) 13–42	39	2.993	0.002	0.935
Section A		4.90 (1.83) 1–8	3.29 (2.43) 0–7	39	2.391	0.011	0.747
Section B		9.30 (3.21) 3–12	7.14 (3.07) 0–12	39	2.198	0.017	0.687
Section C		12.70 (3.92) 0–16	12.71 (2.83) 6–15	39	0.013	0.495	0.004
Section D		7.80 (4.18) 0–15	6.14 (2.22) 3–9	39	1.576	0.063	0.499
Section E		4.40 (3.68) 0–11	2.05 (3.01) 0–8	39	2.247	0.015	0.702

Section A: airway, Section B: breathing, Section C: circulation, D: disability, PS: Performance Score, INT: intervention group, CON: control group, df: degrees of freedom

A total of six items were not addressed in over 70% of all scenarios of both groups (treatment of seizures: 88% (percentage of scenarios item not addressed), use of muscle relaxants: 85%, documentation of the Glasgow Coma Scale: 83%, decision on indication for i.v. thrombolysis: 80%, undressing: 80%, orienting physical examination: 73%). The topics: seizure, Glasgow Coma Scale, and thrombolysis were discussed only in the INT group.

### Assessment of time management and effective use of treatment time

The average time of use of the checklist (CU) in the INT was 6.32 min (2.39–9.18 min; SD = 2.08 min). No correlation found between CU and PS (*r* = 0.255, *p* = 0.277).

In the INT, significantly more items were completed per minute of scenario duration (INT, 1.48 items/min; CON, 1.15 items/min, difference: 0.33/min (29%), *p* = 0.001), showing a large effect size (*d* = 1.11).

### Assessment of degree of organization of the work process

**Fig. 4:**
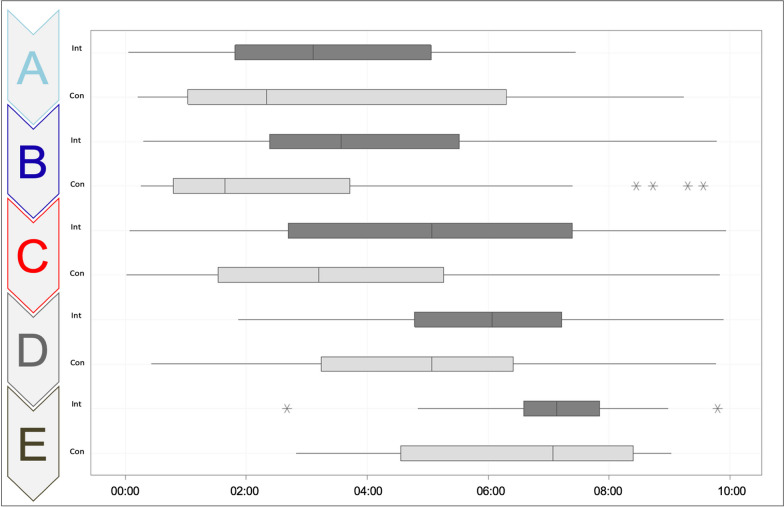
Workflow as a function of time in a graphical form. X-axis: time in min., Y-axis: ABCDE mnemonic as structure of the checklist, Int: intervention group, Con: control group, star: deviating data values

The workflow did not significantly differ between the groups (*p* = 0.079), although a medium effect size was shown (*d* = 0.563) with the tendency of the CON group deviating stronger from the scheme than the INT. Figure 4 shows the workflow as a function of time in a graphical form.

A final reassessment, e.g. in the form of a timeout at the end of ROSC therapy (sum-up), was performed by nine teams in the INT (45%) and none of the CON teams.

The separately evaluated parameter TTM was discussed by 9 out of 20 teams in INT compared to 5 out of 21 CON teams. This difference was not statistically significant (*p* = 0.153). In the post-scenario questionnaire, 64% of the participants in the INT and 75% in the CON rated a TTM for a ROSC patient like in the scenario as ‘*useful to very useful*’ on a 6-point Likert scale (INT: 5 = 23, 6 = 35; n = 90; CON: 5 = 24, 6 = 36; n = 80).

### Additional findings on checklist use

Three-quarters of the INT teams spontaneously adopted the ‘do verify’ approach to checklist usage, with 15% of the teams using it in the form of a ‘challenge-response’ list. The rest of the teams showed a mixed usage (10%). A relevant proportion in both groups interrupted checklist use and repeatedly continued checklist use by repeatedly picking up the list (70%, n = 14). The style of checklist usage (‘do verify’: 38.53 ± 6.59 vs. ‘challenge response’: 40.80) did not show a significantly different PS (*p* = 0.762, *d* = 0.24).

In the pre-scenario questionnaire, attitudes towards the usefulness of checklists in the prehospital setting showed no differences on a 1–6 Likert scale (INT: 4.75 ± 1.25; CON: 4.89 ± 1.17; *p* = 0.167, *d* = 0.122).

In the post-scenario questionnaire, INT rated the effect of checklist use on the duration of care on a 1 (not extended) to 6 (relevant extended) Likert scale as medium (3.57 ± 1.57). The workflow disturbance by the checklist was rated similarly (3.27 ± 1.49). However, the ABCDE structure of the checklist subjectively helped in keeping treatment priorities in the right order (Likert scale 1–6: 4.34 ± 1.22) and not missing out on treatment items (Likert scale 1–6: 4.62 ± 1.38). Most users found the language and terminology of the checklist to be unambiguous (79.0%, n = 73/92).

## Discussion

In the prehospital setting, a 25-item checklist for the management of a patient with ROSC can significantly increase guideline adherence and thereby have a direct, potential positive impact on the outcome. This effect was achieved without the need to invest in additional process time. Rather, the checklist significantly increases the efficient usage of the prehospital resource “time” by enabling a team to complete 29% more tasks per minute.

The ‘ABCDE’ mnemonic, which is established in patient care, can be used as a basic content framework to design a prehospital, guideline-oriented checklist. As a part of the standardized trained prehospital trauma life support [PHTLS]-format the ‘ABCDE’ mnemonic improved efficiency in prehospital care by significantly reducing both transfer times (9.3 vs. 10.5 min, *p* = 0.006) and on-scene time of the teams (36.2 vs. 42.6 min, *p* = 0.003) in an European metropolitan area. [5, 7, 26].

Guideline compliance was measured using a self-defined performance score (PS) Although the global PS differed significantly, individual areas of the ‘ABCDE’ mnemonic showed no difference between the groups. Since the individual subsections mainly consist of items representing established measures of daily routine like the electrocardiogram and blood pressure measurements [6–8, 26]. The checklist could be optimized and simplified by omitting those items. Doing so, acceptance of the checklist can be improved by empowering the provider to use common sense [9].

The checklist with the underlying ‘ABCDE’ mnemonic did not significantly change the workflow of the teams. The analysis showed a medium effect size, indicating that a larger sample size could have resulted in a statistically significant difference, as it is described in the literature [27, 28] More research is needed concerning this question.

The effects differ between the respective parts of the checklist. In our study, checklist use had a more formative effect on A, B, and E than on C and D, illustrating that the ‘ABCDE’ mnemonic is sufficiently established in the ambulance service. The checklist is used as a classic ‘memory aid’, which makes teamwork more efficient and faster [2, 5, 21]. Trained prehospital teams show a parallelization in the processing of tasks, which is potentially negatively influenced by a rigid checklist construct. The defined priority of the checklist (e.g. efficiency of care and guideline adherence) may create a conflict with existing routines [29]. In order to further improve the compliance and the flow during checklist use communication of the team is paramount. The intuitive way of checklist uses of most of the prehospital teams (75%) corresponds to the ‘do verify/ confirm’ form of execution. The tasks are mainly performed from memory and the serves as memory aid to confirm adherence to guidelines [30]. Aiming for resilient checklist use and bracing against interruptions a dynamic flow of authority between team members, independent of hierarchical positions, seems mandatory while using the checklist [9, 29]. E.g. the operator of the checklist may dynamically change between team members.

The target temperature measure (TTM) as a surrogate parameter for accelerated knowledge transfer showed no significant difference between groups with only 45% of groups performing TTM at best in the INT group. The checklist alone was unable to accelerate transfer of knowledge from scientific findings to healthcare practice. Transfer currently lasts at least 17 years [31]. However, current developments, such as the coronavirus disease pandemic, demonstrated the need for tools to rapidly change treatment practice. A well familiarized checklist may address this problem serving as a memory aid.

### Limitation

A simulation can only try to reproduce the care practice as best as possible. Yet, a deviation from reality remains, and therefore, certain findings cannot be intuitively ascertained but require an inquiry. The study participants in both groups rated the realism of the scenario design as high. To allow a reproducible study performance, the supply time was limited to 10 min in both groups. This time interval is realistic for the prehospital setting before transport measures are undertaken. The teams (provider) were not involved in creating the design and content of the checklist. The checklist was only demonstrated as a concept in a short lecture to the participants. The medical content of the checklist (ROSC) was not taught; therefore, the findings of this study must be described as a minimal possible effect of checklist use. In reality, a structured implementation of the checklist with a detailed team training is recommended to maximize the benefit.

The medical guideline referred to in the study is outdated. Valid guidelines reprioritized the relevance of TTM. Since the study had the focus on checklist use and not the medical content of the checklist results of the study are not diminished.

## Conclusion

Checklists can have positive effects on outcome in the prehospital setting by significantly increasing adherence to guidelines. In this context, checklist use is time effective. To maximize these effects, it is recommended that guideline content for checklist use be transferred to the ABCDE mnemonic. The prehospital use of checklists can primarily be performed according to the ‘do verify’ approach. To increase patient safety, a team timeout is recommended at beginning and end of checklists. Local adaptation of checklists enables the reduction of content and increases compliance.

## Data Availability

Please contact author for data requests.
